# Metabolic Reprogramming in Autosomal Dominant Polycystic Kidney Disease: Role in Cystogenesis and Novel Therapeutic Approaches

**DOI:** 10.3390/biomedicines13071596

**Published:** 2025-06-30

**Authors:** Jingyuan Gao, Xiaoyong Yu

**Affiliations:** Nephrology Department, Shaanxi Provincial Hospital of Traditional Chinese Medicine, Xi’an 710003, China; gaojingyuan0820@126.com

**Keywords:** ADPKD, autosomal dominant polycystic kidney disease, metabolic reprogramming, glycolysis, mitochondrial dysfunction, fatty acid oxidation, glutamine metabolism, novel therapeutic

## Abstract

Autosomal dominant polycystic kidney disease (ADPKD) is a prevalent hereditary renal disorder characterized by the progressive formation of numerous fluid-filled cysts, ultimately leading to end-stage kidney disease. The results of recent studies have demonstrated that metabolic reprogramming plays a crucial role in cystogenesis and disease progression, including enhanced aerobic glycolysis, impaired fatty acid oxidation, glutamine dependence, and mitochondrial dysfunction; these metabolic alterations are regulated by signaling pathways such as mTOR, cAMP/PKA, and HIF-1α, which can modulate cell proliferation, fluid secretion, and energy metabolism. Furthermore, hypoxia and the oxidative microenvironment also promote the growth of cysts. In this review, we summarized the complex interactions between metabolic pathway alterations and key signaling cascades in ADPKD, in addition to exploring new therapeutic strategies targeting these metabolic pathways, including drug and dietary interventions. A comprehensive understanding of these mechanisms may contribute to the development of innovative treatment methods aiming to slow the disease progression of patients with ADPKD.

## 1. Introduction

Autosomal dominant polycystic kidney disease (ADPKD) is the most prevalent monogenic kidney disease, affecting around one in every 400–1000 people worldwide [1]. ADPKD is characterized by the development of numerous fluid-filled cysts in both kidneys; the cysts enlarge over time, distorting the renal architecture [2]. Renal function often remains preserved during early adulthood; however, a progressive decline in GFR follows, often leading to end-stage renal disease (ESRD) by the end of middle age [3].

ADPKD results from heterozygous mutations of either the *PKD1* or *PKD2* gene, with *PKD1* mutations being the more prevalent [4,5]. PC1 is a large transmembrane glycoprotein with multiple extracellular domains and a cytoplasmic C-terminal tail that interacts with heterotrimeric G-proteins, initiating signaling cascades such as the JNK AP-1 axis that regulates cell proliferation and apoptosis [6,7,8,9,10]. PC2, also known as TRPP2, is a calcium-permeable nonselective cation channel. In the absence of PC1, PC2 forms a homomeric tetramer, involved in calcium signaling, from the endoplasmic reticulum [11,12,13,14]; in the presence of PC1, PC2 associates with PC1 to form a heteromeric ion channel receptor complex with a 3:1 stoichiometry (3 PC2:1 PC1), functioning as a mechanosensitive ion channel in the primary cilium and regulating calcium influx in response to mechanical stimuli [15,16,17,18]. Mutations in either PC1 or PC2 lead to impaired Ca^2+^ release from endoplasmic reticulum (ER) stores, reducing cytoplasmic Ca^2+^ levels and ultimately disrupting cellular Ca^2+^ homeostasis [19,20,21]. The classical hypothesis for cyst initiation is that, in addition to a germline inactivating mutation in one allele of the *PKD* gene, there is somatic inactivation (referred to as the second hit) in the other allele, causing a complete loss of polycystin expression in the cell [5]; this dysfunction disrupts calcium regulation, altering cell polarity, proliferation, apoptosis, and extracellular matrix remodeling, and activating several signaling pathways, including cAMP, MAPK, mTOR, and canonical Wnt pathways [22,23,24].

Emerging evidence points to metabolic reprogramming as a key contributor to ADPKD pathogenesis. In recent studies, researchers have shown that cyst-lining cells undergo shifts in energy metabolism reminiscent of the Warburg effect observed in cancer cells; ADPKD cyst cells adopt a metabolic phenotype similar to rapidly dividing tumor cells, which helps sustain their continued proliferation and cyst expansion.

Recent work has further expanded understanding of the role of polycystins beyond the kidney. Loss of PC1 or PC2 in vascular endothelial cells impairs flow sensing and disrupts vasodilatory responses, contributing to the early development of hypertension in ADPKD patients, even before significant renal dysfunction occurs. Specifically, polycystin-2 functions as a flow-sensitive calcium channel in endothelial cells, promoting nitric oxide production and vasodilation, while polycystin-1 interacts with PC2 at the plasma membrane to form a receptor–channel complex that transduces mechanical signals into vascular relaxation. Deficiency of either protein compromises these endothelial functions, providing a mechanistic explanation for the prevalence of hypertension in ADPKD independent of renal cyst burden [25,26,27]. Additionally, ADPKD is associated with a range of extrarenal manifestations, including hepatic cysts, intracranial aneurysms, metabolic syndrome, and cardiovascular complications, all of which increase patient morbidity and mortality [25].

Despite these diverse clinical features, current treatment options for ADPKD remain limited. Treatments for ADPKD have been largely supportive to date, revolving around the aggressive control of blood pressure, as well as protective measures for kidney function [28]. In particular, tolvaptan, a vasopressin V2 receptor antagonist, has become the first approved disease-modifying treatment for ADPKD. Tolvaptan inhibits cyclic adenosine monophosphate (cAMP) signaling in the kidney, thereby modestly slowing cyst growth and the decline in kidney function in affected patients [29]. However, the burdens of disease progression and comorbidity in ADPKD underscore the need for new therapeutic strategies grounded in an improved understanding of disease mechanisms.

In this review, we will delve into the mechanistic links between metabolism and cyst growth in ADPKD, evaluate current and emerging metabolic interventions (from dietary modulation to novel drugs), and highlight future directions and unanswered questions in this evolving field.

## 2. Metabolic Pathways Altered in ADPKD

### 2.1. Aerobic Glycolysis and Glucose Metabolism in Cystic Cells

The kidney is a metabolically active organ with high energy demands [30]. In ADPKD, cystic epithelial cells undergo a metabolic shift from oxidative phosphorylation to aerobic glycolysis, resembling the Warburg effect observed in cancer cells [31]; this shift enhances glucose uptake and promotes lactate production despite adequate oxygen availability. Research has shown that *PKD1*-deficient mouse models exhibit significant metabolic alterations, including enhanced glycolysis and lactate production despite oxygen sufficiency, indicating a dependency on aerobic glycolysis. Comprehensive metabolomic analyses have shown that *PKD1*-mutated cells preferentially use glutamine to fuel the tricarboxylic acid (TCA) cycle and facilitate fatty acid synthesis [32].

Glycolytic reprogramming in ADPKD is associated with reduced AMP-activated protein kinase (AMPK) activity. At the same time, the mTOR signaling pathway becomes increasingly active, particularly mTOR complex 1 (mTORC1) and the transcription factor c-Myc, both of which are generally upregulated in ADPKD [33]. mTORC1 is a key regulator of cell growth and metabolism. In ADPKD, hyperactivation of mTORC1 drives glycolysis by increasing the expression of key glycolytic enzymes [34]; it also upregulates hypoxia-inducible factor 1-alpha (HIF-1α), which further promotes the transcription of genes involved in glycolysis [35].

Glycolytic reprogramming in ADPKD is associated with reduced AMP-activated protein kinase (AMPK) activity. At the same time, the mTOR signaling pathway becomes increasingly active, particularly mTOR complex 1 (mTORC1) and the transcription factor c-Myc, both of which are generally upregulated in ADPKD. mTORC1 is a key regulator of cell growth and metabolism. In ADPKD, hyperactivation of mTORC1 drives glycolysis by increasing the expression of key glycolytic enzymes, and it upregulates hypoxia-inducible factor 1-alpha (HIF-1α), which further promotes the transcription of genes involved in glycolysis; these changes contribute to the metabolic reprogramming seen in cystic epithelial cells.

c-Myc, a transcription factor involved in cell growth and metabolic regulation, is similarly upregulated within ADPKD; it directly promotes the transcription of central glycolytic enzymes hexokinase 2 (HK2) and lactate dehydrogenase A (LDHA), thus promoting the conversion of glucose to lactate; this metabolic adaptation not only promotes energy production but also provides the biosynthetic intermediates necessary for the rapid growth of cells [36]. mTORC1 enhances c-Myc expression, while c-Myc further activates mTORC1, leading to sustained metabolic reprogramming [37].

Similar to tumor cells, cystic epithelial cells can cause lactic acidosis through their dependence on aerobic glycolysis [38]. Lactic acidosis in tumors has been shown to contribute to resistance against the cell death caused by glucose deprivation. By analogy, such a liability may be present in ADPKD, making the inhibition of glycolysis a potential therapeutic strategy [39]. In preclinical studies, researchers have demonstrated that glycolytic inhibition attenuates cyst growth in ADPKD models [40]. Treatments with substances such as 2-deoxyglucose (2-DG) and metformin have been noted to inhibit the growth of cystic epithelial cells synergistically through the interference of glucose metabolism, thereby reestablishing cellular energy homeostasis [38].

### 2.2. Mitochondrial Dysfunction and Oxidative Phosphorylation

Mitochondrial dysfunction is one of the core features of ADPKD, with a pivotal role in the disease’s pathogenesis. Healthy renal tubular epithelial cells rely on the mitochondria for ATP generation through oxidative phosphorylation (OXPHOS) [4]. However, in ADPKD, cystic epithelial cells exhibit impaired mitochondrial function, leading to a shift toward aerobic glycolysis as the primary energy source [41]. ADPKD was found to have a global downregulation of transcription of those involved in mitochondrial oxidative metabolism, including the TCA cycle and the electron transport chain, leading to decreased OXPHOS capacity and impaired fatty acid oxidation (FAO) and, further, to the over-reliance of cells on a less efficient glycolysis process through which to generate ATP; this metabolic transformation provides energy for the proliferation of cystic epithelial cells and promotes the production of reactive oxygen species (ROS) [4].

One of the pivotal molecular drivers of this mitochondrial impairment is the loss of PC1 function. In recent studies, researchers have elucidated that the C-terminal tail of PC1 interacts directly with the mitochondrial enzyme nicotinamide nucleotide transhydrogenase (NNT), a key modulator of redox balance; this interaction has been shown to suppress cyst growth by enhancing mitochondrial antioxidant defense mechanisms and preserving mitochondrial function. Onuchic et al. demonstrated that truncation of PC1’s C-terminal tail in a *PKD1* mutant mouse model leads to impaired NNT function, elevated oxidative stress, and more aggressive cyst progression. Restoration of this interaction normalized mitochondrial redox homeostasis and significantly mitigated disease severity [42].

Further evidence implicates that the transcription factor GLIS3 is involved in mitochondrial biogenesis and function. In a recent study, researchers found that GLIS3 deficiency in kidney cells suppresses genes essential for mitochondrial function, increases reliance on aerobic glycolysis, and reduces OXPHOS activity [43].

The outcomes of many studies have shown that the integrity of mitochondria plays an important role in maintaining normal renal function and preventing cyst formation. For instance, in the ADPKD mouse model, a decline in mitochondrial mass and structural changes in cystic cells were observed [44]. Cassina et al. further demonstrated that mitochondrial fragmentation and reduced mass in *PKD1* mutation-based models contribute to these pathological changes [45].

Mitochondrial dysfunction in ADPKD leads to a state of pseudohypoxia within renal cysts. As cysts enlarge, they can outgrow their blood supply, creating a localized hypoxic environment, compounded by mitochondrial impairment, which stabilizes hypoxia-inducible factor 1-alpha (HIF-1α) in cyst-lining cells, promoting glycolysis and angiogenesis, thereby accelerating cyst growth in a feed-forward loop. Moreover, defective mitochondria generate excess ROS, contributing to oxidative stress and further promoting cystogenesis. Ishimoto et al. observed mitochondrial abnormalities in ADPKD, including a decrease in mitochondrial DNA copy number and suppressed expression of peroxisome proliferator-activated receptor γ coactivator 1α (PGC-1α), leading to increased oxidative stress and HIF-1α stabilization in cystic epithelial cells [44].

Pseudohypoxia, characterized by the stabilization of HIF-1α under normoxic conditions, has been implicated in the pathogenesis of renal cysts. The accumulation of metabolic intermediates and ROS inhibits prolyl hydroxylases, preventing HIF-1α degradation and promoting its transcriptional activity [46]. Korsmo et al. emphasized that ROS overproduction, primarily from dysfunctional mitochondria and NADPH oxidase 4 (NOX4), leads to oxidative damage to mitochondrial DNA, proteins, and lipids, impairing cellular function and exacerbating cyst progression [47].

Daneshgar et al. further highlighted the relationship among oxidative stress, mitochondrial dysfunction, and metabolic derangement in *PKD1* mutant models, demonstrating a downregulation of TCA cycle enzymes and mitochondrial respiratory complex activity that contributes to a glycolytic shift and cyst expansion [48]. Kahveci et al. reported that early-stage PKD is associated with NOX4-induced oxidative stress and mitochondrial abnormalities in endothelial cells, leading to endothelial dysfunction, capillary loss, and the exacerbation of renal disease [49].

Therapeutic strategies targeting mitochondrial dysfunction have shown promise. Daneshgar et al. observed that overexpression of mitochondrial-targeted catalase (mCAT) reduced mitochondrial ROS levels, alleviated oxidative damage, and attenuated cyst progression in *PKD1* mutant models [48]. Furthermore, treatment with SS31, a mitochondrial protective peptide, recapitulated the beneficial effects of mCAT, indicating the therapeutic potential of targeting mitochondrial oxidative stress in ADPKD. Mitochondrial antioxidants, such as SS31, have also been shown to slow cyst growth and reduce fibrosis in *PKD1*^RC/RC^ mouse kidneys, supporting the potential of mitochondrial-targeted therapies in managing ADPKD [50].

The activation of signaling pathways, such as mTORC1 and c-Myc, plays a significant role in metabolic reprogramming by further suppressing oxidative metabolism and simultaneously promoting glycolytic activity; these pathways inhibit mitochondrial biogenesis and function, contributing to the metabolic alterations observed in cystic epithelial cells. Preclinical models have shown that restoring mitochondrial function through the activation of AMPK or PGC-1α improves mitochondrial biogenesis and reduces cyst growth [4].

### 2.3. Altered Lipid Metabolism and Fatty Acid Oxidation

In ADPKD, cyst-lining epithelial cells undergo a significant transformation; they no longer use FAO as the primary energy source [51]. The sign of this transformation is the significant downregulation of the proteins involved in FAO in cystic kidneys [52]. Key enzymes, such as carnitine palmitoyltransferase 1 (CPT1) and acyl-CoA dehydrogenases (ACADs), are suppressed, a result which suggests a decrease in mitochondrial fatty acid oxidation capacity [48,53].

Ketone body utilization relies on enzymes, including 3-hydroxybutyrate dehydrogenase (BDH1) and succinyl-CoA:3-ketoacid CoA transferase (SCOT, encoded by OXCT1), which are part of lipid and mitochondrial metabolism pathways [51]. The authors of some studies have found that those pathways are downregulated, suggesting that ketolysis may be impaired. Moreover, cystic epithelial cells in ADPKD show a diminished capacity to use ketone bodies as an alternative source of energy [4]. Therefore, therapeutic intervention measures, such as ketogenic diet and exogenous ketone supplementation, are used to increase overall ketone levels. The aforementioned approaches have demonstrated potential in slowing disease progression [54].

In ADPKD, the diversion of citrate toward lipid biosynthesis is increased. *PKD1* mutant cells also showed an upregulation of fatty acid synthase (FAS) expression [32]. The activation of mTORC1 upregulates key transcription factors, such as HIF-1α and c-Myc [55], which increase cellular sugar metabolism by promoting the representation of the *GLUT1*, *HK2*, *PFK1*, and *PKM2* genes [56]. Furthermore, mTORC1 promotes the expression of enzymes involved in fatty acid synthesis, such as ACC, FAS, and SCD1 [57] and provides crucial precursors for the rapidly growing cytomembrane [58].

There is a link among fat, metabolic syndrome, and the accelerated development of ADPKD, according to scientific research [59,60]. A faster decline in renal function, as well as a higher body mass index (BMI) and visceral adiposity, are related to a larger total kidney volume (TKV) [61].

Elevated insulin levels and nutrient availability further enhance hormonal metabolic processes, promoting cyst growth. The activation of mTORC1 by insulin and nutrient signals also enhances the expression of glycolytic and lipogenic genes, thereby supporting cyst expansion [4]. Peroxisome proliferator-activated sensor alpha (PPARα), a central regulator of FAO, plays a vital role in this process. In experimental models of ADPKD, the activation of PPARα has been shown to reduce cyst formation. Importantly, treatment with PPARα receptors, such as fenofibrate, increases FAO and slows disease development in *PKD1*^RC/RC^ mice [62].

### 2.4. Amino Acid Metabolism and Autophagy

In ADPKD, cystic epithelial cells undergo intensive metabolic programming to meet their biochemical and regenerative needs, resulting in significant amino acid digestion changes. In one study, researchers found that supplementing *PKD1*-deficient mice with branched-chain amino acids (BCAAs), especially leucine, led to increased cystic indices in both the kidneys and liver. The observed effect was mediated by the activation of the mTOR and MAPK/ERK signaling pathways, which, in turn, promoted the proliferation of cyst-lining cells [63].

Among the key metabolic alterations, glutamine emerges as a crucial fuel for *PKD1*-deficient cells, which exhibit increased glutamine consumption, using it as an anaplerotic substrate for the TCA cycle and as a nitrogen donor for biosynthesis. Glutamine is converted via glutaminolysis into α-ketoglutarate, which feeds into a truncated TCA cycle to generate ATP and biosynthetic precursors. Podrini et al. observed that *PKD1* mutant cells preferentially utilize glutamine to fuel the TCA cycle and sustain FAS; additionally, they found that glutamine is diverted to asparagine through enzyme asparagine synthetase (ASNS) [32].

Asparagine levels are significantly elevated in ADPKD patient plasma, with more than a threefold increase observed even in the early stages of the disease [64]; this metabolic alteration has drawn parallels to the metabolic shifts observed in cancer cells, where glutamine metabolism is similarly reprogrammed.

Glutaminase inhibitors, which target this altered glutamine metabolism, are being explored in clinical trials as therapeutic options for various cancers. In ADPKD, treatment with a glutaminase inhibitor in *PKD1*-mutant mice, both in utero and postnatally, resulted in slowed cyst progression, highlighting the potential therapeutic benefit of targeting glutamine metabolism in ADPKD [40].

Autophagy is a vital cellular mechanism that degrades and recycles damaged organelles and proteins, thereby maintaining cellular homeostasis. Emerging evidence suggests that dysregulated autophagy contributes significantly to the progression of ADPKD. Autophagy abnormalities are marked by decreased autophagosome formation, as well as the accumulation of autophagy-related proteins such as p62/SQSTM1. In preclinical models, it has been demonstrated that pharmacological agents, such as trehalose and carbamazepine, unlock autophagy; by promoting the clearance of damaged mitochondria and limiting epithelial cell proliferation, these agents help suppress cyst formation [65].

Autophagy has a variety of roles in ADPKD. On the one hand, promoting autophagy may stop the spread of the condition by preventing the deterioration of cells and reducing oxidative stress. On the other hand, overwhelming or dysregulated autophagy may unintentionally promote the development and survival of severe epithelial tissues; therefore, reestablishing proper autophagic activity may help alleviate mitochondrial dysfunction and decrease cyst burden, an approach that presents a promising strategy for slowing the progression of ADPKD.

### 2.5. Redox Imbalance and Cellular Microenvironment

In ADPKD, metabolic reprogramming leads to increased glycolysis and impaired mitochondrial function, resulting in an elevated production of ROS and weakened antioxidant defenses; for example, the NRF2 pathway, a key regulator of antioxidant responses, is suppressed due to the accelerated degradation of NRF2 protein, exacerbating oxidative stress, which, in turn, accelerates the initiation and progression of renal cysts. The pharmacological activation of NRF2 has been shown to reduce ROS levels and slow cyst formation in mouse models, a finding which suggests that targeting this pathway could be a promising approach to slowing disease progression in ADPKD patients [66].

In experimental studies, it has been shown that the pharmacological activation of NRF2 reduces ROS production and slows cyst growth in mouse models, highlighting the therapeutic potential of targeting the NRF2 pathway to mitigate disease progression in ADPKD patients [66].

Metabolic reprogramming also disrupts cellular energy balance by enhancing glycolysis and impairing mitochondrial respiration, thereby amplifying ROS accumulation and diminishing antioxidant defenses [67].

Research has shown that, as cysts in ADPKD expand, they exert pressure on the surrounding renal tissue and blood vessels; this compression results in localized hypoxia within the affected area, stabilizing HIF-1α in cystic epithelial cells, which then activates genes that facilitate cyst growth, including those involved in fluid secretion and cell proliferation; for instance, Kraus et al. showed that the deletion of HIF-1α in a *PKD1*-deficient mouse model resulted in reduced cyst growth, highlighting the role of HIF-1α in cyst progression [68].

Additionally, HIF can decrease fatty acid oxidation and mitochondrial function, reinforcing the metabolic shift. Menezes et al. reported that cells lacking *PKD1* have an intrinsic fatty acid oxidation defect [52]. The results of some studies have demonstrated elevated levels of HIF-1α and HIF-2α in both human and mouse models of ADPKD [69].

Regarding the adverse effects of PHD inhibitors in ADPKD models, the authors of experimental studies using PHD inhibitors, which stabilize HIF, have reported aggravated cyst progression in ADPKD mouse models [70,71]. The administration of these inhibitors led to the rapid loss of renal function and increased cyst growth, suggesting that HIF stabilization may be detrimental in the context of ADPKD.

**Figure 1:**
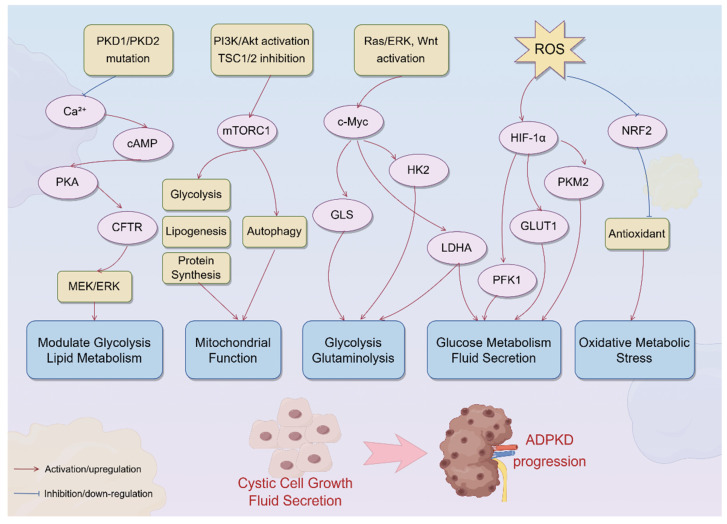
Signaling pathways driving metabolic reprogramming in ADPKD. The schematic summarizes the major signaling pathways involved in metabolic reprogramming in ADPKD. The loss of polycystin function due to *PKD1/PKD2* mutations disrupts primary ciliary calcium signaling, resulting in cAMP accumulation and protein kinase A (PKA) activation. PKA, through the downstream activation of CFTR and the MEK/ERK pathway, promotes fluid secretion and epithelial cell proliferation, while modulating glucose and lipid metabolism. Concurrently, PI3K/Akt pathway activation and TSC1/2 complex inhibition lead to mTORC1 hyperactivation, which enhances glycolysis, lipogenesis, and protein synthesis and inhibits autophagy, ultimately impairing mitochondrial function. Activation of the Ras/ERK and Wnt pathways induces c-Myc, which upregulates key metabolic enzymes, such as hexokinase 2 (HK2), lactate dehydrogenase A (LDHA), and glutaminase (GLS), driving aerobic glycolysis and glutaminolysis. Mitochondrial dysfunction and increased ROS production stabilize HIF-1α, further promoting glycolytic flux via GLUT1, HK2, PFK1, PKM2, and LDHA upregulation. An excess of ROS also accelerates the degradation of NRF2, weakening antioxidant defense and exacerbating oxidative metabolic stress; these converging pathways orchestrate a profound shift in metabolic programming, fueling cystic cell growth, fluid secretion, and disease progression in ADPKD. PKA, protein kinase A; CFTR, cystic fibrosis transmembrane conductance regulator; ERK, extracellular signal-regulated kinase; PI3K, phosphoinositide 3-kinase; mTORC1, mechanistic target of rapamycin complex 1; TSC1/2, tuberous sclerosis complexes 1 and 2; HK2, hexokinase 2; LDHA, lactate dehydrogenase A; GLS, glutaminase; ROS, reactive oxygen species; HIF-1α, hypoxia-inducible factor 1-alpha; NRF2, nuclear factor erythroid 2-related factor 2.

Multiple signaling pathways converge to orchestrate the profound metabolic reprogramming observed in cystic epithelial cells in ADPKD, including the cAMP/PKA axis, PI3K/Akt/mTORC1 signaling, Ras/ERK-induced c-Myc activation, mitochondrial dysfunction–induced HIF-1α stabilization, and NRF2 suppression. A mechanistic overview is illustrated in Figure 1, which summarizes the cascade from *PKD1/2* mutations to the downstream metabolic alterations that sustain cyst growth and fluid secretion.

## 3. Molecular Mechanisms Linking Metabolism to Cystogenesis

### 3.1. cAMP/PKA Pathway

As discussed in the Introduction, mutations in PC1 or PC2 reduce cytoplasmic Ca^2+^ levels and, ultimately, disrupt cellular Ca^2+^ homeostasis [19]. Lower calcium concentrations diminish the inhibitory effect on adenylyl cyclases (ACs), particularly AC5 and AC6, resulting in increased cAMP synthesis [72]. Additionally, decreased calcium levels impair calcium-dependent phosphodiesterases (PDEs), such as PDE1, reducing cAMP degradation and further contributing to its accumulation [73]. Elevated cAMP levels in ADPKD also drive transepithelial fluid secretion into cyst lumens. cAMP activates the cystic fibrosis transmembrane conductance regulator (CFTR) chloride channel, facilitating chloride ion secretion into the cyst lumen [74]; this ion movement creates an osmotic gradient that draws water into the cysts, leading to their expansion. Researchers have shown that increased levels of cAMP within ADPKD activate PKA, which subsequently leads to the phosphorylation of CREB, as well as that of CRTC2 [75].

Reducing intracellular calcium relieves Akt from inhibiting B-Raf, enabling the activation of cAMP to activate B-Raf, which results in the activation of the MEK/ERK pathway, promoting cell growth and leading to the formation of cysts [74].

The results of preclinical and clinical studies of cystic models have shown that reducing cAMP levels with tolvaptan or somatostatin analogs can normalize the expression of metabolic genes [76]. Strong evidence supports the involvement of cAMP signaling in cystogenesis; however, several reports suggest a more complex relationship between cAMP levels and the expression of metabolic genes. For example, there are some models in which decreasing cAMP levels has been shown to fail to fully normalize the expression of metabolic genes [77], suggesting that there are other pathways involved in the development of metabolic derangements of ADPKD.

### 3.2. mTOR and AMPK

Decreased intracellular calcium concentrations lead to reduced inhibition of PI3K and promote Akt activation [78]. Moreover, the loss of functional PC1 results in increased activation of the Ras/Raf pathway with stimulation of IGF-1, leading to PI3K/Akt pathway activation [79]. Activated Akt promotes cell growth and survival through the phosphorylation of downstream growth and apoptosis inhibitory targets involved in cyst growth and expansion [80]. The PI3K/Akt pathway does not exist independently; it interacts with other signaling mechanisms implicated in ADPKD pathogenesis, and Akt activation can influence the mTOR pathway.

The mTOR pathway is a key controller of cell growth, proliferation, and survival. Aberrant activation of mTOR has been found both in the disease model of ADPKD and in humans [81], but the mechanisms of mTOR hyperactivation leading to ADPKD are complex. The activation of mTORC1 has been shown to result in the increased expression of enzymes involved in glycolysis, such as HK2 and pyruvate kinase [82]; this upregulation occurs through HIF-1α, which is stabilized under mTORC1 activation [4]. In models of kidney fibrosis, deletion of tuberous sclerosis complex 1 (Tsc1), a negative regulator of mTORC1, resulted in enhanced glycolysis and tubular epithelial cell proliferation [83].

mTORC1 activation stimulates anabolic pathways, including protein and lipid biosynthesis, which is achieved through the activation of transcription factors such as sterol regulatory element-binding proteins (SREBPs) and HIF-1α, which drive the expression of genes involved in lipid and protein synthesis [84]. Researchers have demonstrated that, in ADPKD models, there is a notable decrease in AMPK activity, accompanied by increased mTORC1 signaling [85]. The reduced activity of AMPK in ADPKD leads to the decreased expression of peroxisome PGC-1α, a key regulator of mitochondrial biogenesis [85]. Consequently, there is a decline in mitochondrial number and function, impairing oxidative phosphorylation and increasing the reliance on glycolysis. The pharmacological activation of AMPK, using agents such as metformin and AICAR, has shown promise in ADPKD preclinical models. The results of one study showed that metformin treatment in a slowly progressive ADPKD mouse model led to reductions in kidney weight-to-body weight ratio, cystic index, and blood urea nitrogen levels. Additionally, metformin improved glomerular filtration rate, lowered systolic blood pressure, ameliorated anemia, and decreased markers of inflammation and kidney injury [86]. The aforementioned treatments have been associated with reduced mTORC1 activity, enhanced autophagy, improved mitochondrial function, and attenuation of cyst growth; however, in certain models, metformin’s effects on cyst growth were observed even in the absence of significant changes in AMPK activity, indicating potential AMPK-independent mechanisms [86].

### 3.3. c-Myc and Other Oncogenic Signals

The results of some studies have demonstrated that c-Myc expression is elevated in cystic epithelial cells in ADPKD. The loss of polycystin function in ADPKD activates signaling pathways, including the Ras/ERK and Wnt, which can lead to increased c-Myc expression; c-Myc is known to reprogram metabolism by upregulating glycolytic enzymes and glutamine transporters, such as ASCT2, metabolic changes that promote cell proliferation in cystic epithelial cells.

Parrot et al. reported that the expressions of canonical c-Myc metabolic target genes *LDHA*, enolase, and glutaminase (GLS) are significantly elevated in cystic tissues from *PKD1*^KO^ mice [87]. Kraus et al. showed that HIF-1α stabilization in hypoxic cystic microenvironments increases the expression of glycolytic genes, including *LDHA* and *HK2*, mimicking Warburg-like reprogramming [68]; these enzymes are essential for sustaining aerobic glycolysis and glutaminolysis, two core metabolic pathways supporting rapid cell growth. Podrini et al. demonstrated that *PKD1*-deficient cells exhibit enhanced c–Myc–dependent expression of GLS, a glutamine catabolic enzyme, linking glutamine metabolism directly to c-Myc activity [32,88]. Chromatin remodeling enzymes, such as histone deacetylases (HDACs) and bromodomain, and extra-terminal motif (BET) proteins, are involved in ADPKD pathogenesis [89]; these factors regulate the accessibility of transcriptional machinery to metabolic and proliferative gene loci.

### 3.4. Polycystin Signaling and Metabolism

In ADPKD, mutations in the *PKD1* and *PKD2* genes result in the loss of function of PC1 and PC2, respectively; these proteins are localized to the primary cilium and ER, where they play critical roles in calcium (Ca^2+^) signaling [80]. In health, PC1 and PC2 form a complex that functions as a mechanosensor in the primary cilium, mediating Ca^2+^ influx in response to mechanical stimuli [80]; this influx is essential for activating CAMKK2, which subsequently activates AMPK, a key energy sensor that promotes mitochondrial biogenesis and regulates cellular metabolism. Loss of PC1/PC2 disrupts this signaling pathway, leading to decreased AMPK activation and altered metabolic states.

The authors of many studies reported that PC1 hurts the TSC-mTOR pathway [90]; the TSC complex, consisting of proteins TSC1 (also called hamartin) and TSC2 (also called tuberin), plays a role as a negative regulator of mTOR kinase. PC1 has been shown to interact with tuberin (TSC2), a critical inhibitor of mTORC1, and the loss of functional PC1 disrupts this interaction, leading to unchecked mTORC1 activation and subsequent cyst epithelial cell proliferation [90]. Polycystin dysfunction triggers a cascade, such as those in cAMP/PKA, Ca^2+^/AMPK, and mTOR/c-Myc/HIF.

The results of mechanistic studies support that metabolic reprogramming, orchestrated by cAMP, mTOR, and AMPK dysregulation, plays an important role in the pathogenesis of ADPKD. The resulting metabolic phenotype reinforces cystic proliferation through a bidirectional feedback loop involving glutamine dependence, glycolysis, and ROS. However, some debate remains as to whether these changes are causative or secondary phenomena, and therapeutic translation remains a promising area of investigation.

## 4. Therapeutic Approaches Targeting Metabolic Reprogramming

### 4.1. Dietary and Lifestyle Interventions

#### 4.1.1. Caloric Restriction and Fasting

Dietary interventions that reduce energy intake, such as caloric restriction (CR), intermittent fasting (IF), time-restricted feeding (TRF), and ketogenic diets, have demonstrated significant efficacy in preclinical models; these interventions modulate cellular metabolism, particularly by inducing ketosis, which appears to counteract cyst growth and disease progression. The results of some studies have demonstrated that dietary interventions inducing ketosis can significantly inhibit renal cyst growth and preserve kidney function in animal models of ADPKD [91]. Specifically, a study revealed that time-restricted feeding, without reducing caloric intake, led to a marked decrease in mTOR signaling, cell proliferation, and fibrosis in the kidneys of a PKD rat model [92]. Furthermore, ketogenic diets and the administration of β-hydroxybutyrate (BHB) resulted in the regression of renal cystic burden. Kipp et al. demonstrated the feasibility of daily caloric restriction (DCR) and intermittent fasting (IMF) in overweight or obese ADPKD patients. Clinically significant weight loss occurred with both DCR and IMF; however, weight loss was greater and adherence and tolerability were better with DCR. In an orthologous ADPKD mouse model, only animals on DCR lost significant weight and showed slowed cyst growth compared with ad libitum, IMF, or TRF feeding, indicating that weight loss driven by caloric restriction may be beneficial [93]. The above findings suggest that the benefits are attributable to the induction of ketosis, rather than mere caloric restriction, highlighting the metabolic inflexibility of cystic cells as a potential therapeutic target.

The results of mechanistic studies indicate that reducing energy intake through dietary interventions can counter ADPKD progression by modulating metabolism; the induction of ketosis appears to play a pivotal role in this process, as it exploits the metabolic inflexibility of cystic cells, leading to reduced proliferation and cyst growth. While preclinical models show promising results, further clinical research is necessary to establish the safety, efficacy, and feasibility of these interventions in human patients.

Fasting and TRF induce a metabolic shift from glucose utilization to fatty acid oxidation and ketone body production [94]; this transition is characterized by the depletion of hepatic glycogen stores, leading to increased lipolysis and β-oxidation of fatty acids, resulting in elevated levels of ketone bodies such as β-hydroxybutyrate [95]; this metabolic adaptation not only provides an alternative energy source during periods of nutrient scarcity but also modulates key signaling pathways involved in cellular energy homeostasis [95]. One of the critical regulators affected by this metabolic shift is the mTORC1, a central node in cell growth and metabolism. Under conditions of energy deprivation, such as fasting, mTORC1 activity is suppressed, leading to the inhibition of anabolic processes and the activation of catabolic pathways, such as autophagy. AMPK is an energy sensor, activated by increased AMP/ATP ratios [96]. Activated AMPK promotes catabolic processes to generate ATP and inhibits mTORC1 activity, thereby reinforcing the suppression of anabolic pathways during energy stress [97]; this coordinated regulation between mTORC1 inhibition and AMPK activation during fasting and TRF contributes to the restoration of metabolic homeostasis. In the context of kidney physiology, these changes may recreate a more “normal” metabolic state.

In an SPRD rat model of polycystic kidney disease (PKD), TRF without caloric reduction was shown to significantly suppress mTOR signaling, reduce cellular proliferation, and attenuate fibrosis in cystic kidneys. Acute fasting in multiple PKD models (rat, mouse, and even cat) led to rapid reductions in total kidney cyst volume [98]. The above findings suggest that cystic cells cannot easily adapt to ketone-based energy, so their growth stalls or reverses under fasting/ketosis.

#### 4.1.2. Ketogenic Diet and Ketone Supplementation

In preclinical models of ADPKD, ketogenic diets—characterized by high-fat, very low-carbohydrate intake—have been shown to induce a metabolic state of sustained ketosis; this metabolic shift forces the utilization of ketone bodies, such as BHB and acetoacetate, as primary energy sources instead of glucose.

Torres J.A. et al. found that both BHB and citrate effectively halted the progression of PKD in juvenile rats, and their combination led to the partial reversal of existing disease in adult rats; the combination therapy reduced cyst number and area, preserved glomerular health, and decreased markers of kidney injury [99]. BHB activates AMPK, inhibits the mechanistic target of mTORC1, reduces insulin levels, and serves as an efficient energy substrate for non-cystic renal cells [100,101]. In a randomized controlled trial, the feasibility and impact of ketogenic dietary interventions in patients with ADPKD were evaluated; the study results demonstrated that both ketogenic diets and water fasting induced significant ketogenesis, with participants showing reductions in body fat and improvements in eGFR. Post hoc analyses indicated a significant reduction in total kidney volume among individuals with consistent ketone levels, suggesting that diet-induced ketosis may offer therapeutic benefits in ADPKD [102]. While long-term safety and adherence to ketogenic regimens need evaluation, metabolic therapy through diet is a promising low-cost approach that directly targets the metabolic vulnerabilities of ADPKD.

#### 4.1.3. Protein or Amino Acid Restriction

Emerging evidence suggests that the dietary modulation of amino acid intake—specifically protein, glutamine, and arginine—may influence cyst growth in ADPKD. In a study using the DBA/2FG-pcy mouse model of polycystic kidney disease, dietary protein restriction (6% casein) led to a reduction in kidney weight relative to body weight compared with a normal-protein diet (25% casein) [103], which suggests that lower protein intake may attenuate cyst growth in early disease stages.

However, clinical data on protein intake and ADPKD progression are limited. In an observational study with ADPKD, researchers found no significant association between protein intake and the rate of disease progression, as measured via eGFR decline [104]. Research indicates that cystic epithelial cells in ADPKD exhibit a dependency on glutamine metabolism.

The inhibition of glutaminase, the enzyme responsible for converting glutamine to glutamate using the inhibitor BPTES, resulted in a significant reduction in cystic burden in mouse models [40], which suggests that targeting glutamine metabolism may be a viable therapeutic strategy. Arginine restriction also appears to influence cyst growth; in in vitro studies, it has been shown that arginine deprivation can inhibit cyst growth, with *PKD1*-null cell lines exhibiting reduced proliferation under low-arginine conditions [105]. Additionally, the inhibition of arginase-1 (Arg1), an enzyme involved in arginine metabolism, significantly slowed cyst growth and lowered proliferative indices in polycystic kidneys [64].

#### 4.1.4. Hydration and Caffeine

In the management of ADPKD, lifestyle interventions, such as increased water intake and caffeine avoidance, are often recommended as adjunctive measures targeting the vasopressin–cAMP signaling axis. While not classified as direct metabolic therapies, these strategies aim to modulate pathways implicated in cystogenesis and disease progression. Increasing water intake can inhibit the release of endogenous vasopressin, thereby reducing cAMP levels within renal tubular cells [106]. The results of preclinical studies have demonstrated that increased water intake can slow renal cyst growth in animal models via direct vasopressin suppression [107]. In human studies, the PREVENT-ADPKD trial was conducted to compare standard water intake to increased free water intake in 184 participants over three years [108]. However, adherence challenges were noted, with half of the participants in the high-water intake group failing to reach the target urine osmolality, and a third of participants being at low risk for disease progression. The above findings suggest that, while increased water intake is a rational approach, its efficacy may be limited by patient compliance and individual risk profile.

Caffeine, a known phosphodiesterase inhibitor, can increase intracellular cAMP levels. The results of in vitro studies have shown that caffeine stimulates cAMP production in ADPKD and ARPKD cells, leading to increased cellular proliferation [109]; however, clinical data on the impact of caffeine intake on ADPKD progression are limited. In a cross-sectional study involving 102 ADPKD patients and healthy controls, no association between caffeine intake and kidney volume within the range of caffeine consumption was observed. Despite the lack of definitive clinical evidence, avoiding high caffeine intake is generally recommended as a precautionary measure [110].

### 4.2. Pharmacological Metabolic Therapies

#### 4.2.1. AMPK Activators

Metformin, a widely used antidiabetic agent, has garnered attention for its potential therapeutic role in ADPKD, due to its activation of AMPK. The activation of AMPK by metformin is hypothesized to inhibit the mTOR pathway, reduce cystic epithelial cell proliferation, and possibly restore mitochondrial function. The results of preclinical studies showed that metformin slowed cyst expansion and improved kidney function in PKD rodent models. Takiar et al. [111] demonstrated that metformin activates AMPK, leading to the inhibition of both the CFTR and mTOR pathways. Metformin treatment resulted in significant arrest of cystic growth in both in vitro and ex vivo models, as well as a decrease in cystic index in two mouse models of ADPKD.

Through several clinical studies, researchers have assessed the safety, tolerability, and preliminary efficacy of metformin in ADPKD patients. In a retrospective study, Pisani et al. [112] evaluated the effects of metformin on renal function in ADPKD patients with type 2 diabetes mellitus. Over a three-year follow-up, the metformin-treated group exhibited a slower decline in eGFR compared with controls, suggesting a potential renoprotective effect of metformin in ADPKD. The Trial of Administration of Metformin in Polycystic Kidney Disease (TAME-PKD) was a phase 2, randomized, placebo-controlled trial assessing the safety and tolerability of metformin in non-diabetic adults with ADPKD [113]. Over a two-year period, metformin was found to be safe and well tolerated, with a non-significant trend toward a slower decline in eGFR compared to placebo [114]. The conclusions of the study showed that, while metformin is safe for ADPKD patients, larger trials are necessary to evaluate its efficacy in slowing disease progression.

Salsalate, a prodrug of salicylate with established anti-inflammatory properties, has garnered attention for its role as a direct activator of AMPK. In recent preclinical studies, its potential therapeutic effects in ADPKD were explored. Leonhard et al. [115] reported that salsalate administration led to a significant reduction in kidney cyst growth in an adult-onset conditional *PKD1*^KO^ mouse model; the study results suggest that salsalate’s benefits are mediated through AMPK activation. Song et al. [116] evaluated the combined effects of salsalate and tolvaptan in a *PKD1* mutant mouse model; the combination therapy attenuated kidney injury, cell proliferation, inflammation, and fibrosis more effectively than either agent alone, findings which underscore the potential of salsalate, especially in combination therapies, to modulate disease progression in ADPKD. While not yet in large trials, salsalate represents a promising candidate. Overall, by boosting AMPK, these drugs counter metabolic reprogramming: they inhibit mTORC1 and even the CFTR channel responsible for fluid secretion, yielding a dual effect on cyst growth and fluid accumulation.

#### 4.2.2. Glycolysis Inhibition

The glucose analog 2DG, which inhibits glycolysis by being phosphorylated to 2DG-6-phosphate and trapped within cells, has demonstrated efficacy in multiple ADPKD models. Rowe et al. [117] first demonstrated that 2DG dramatically reduced the proliferation of *PKD1*-null cells and slowed cyst growth in vivo. In subsequent studies, researchers confirmed that partial glycolytic inhibition can reduce kidney enlargement without undue toxicity to normal cells (since normal cells can compensate by using mitochondria) [118]. However, the authors caution about the potential systemic side effects of chronic 2DG use in humans. While the chronic administration of 2DG has demonstrated efficacy in preclinical models, its long-term use in humans is constrained by potential side effects, such as hyperglycemia and toxicity [119].

Other inhibitors, such as glucose transporter blockers or lactate dehydrogenase inhibitors, can disrupt the energy supply of these cells, potentially attenuating cyst growth. Phlorizin is a nonselective inhibitor of sodium–glucose cotransporters (SGLTs), which has been shown to induce glycosuria and osmotic diuresis. In the Han: SPRD rat model of PKD, phlorizin treatment resulted in reduced cyst growth and improved renal function [120]. Dapagliflozin is a selective SGLT2 inhibitor that increases glucose excretion and urine output; however, in the PCK rat model of PKD, dapagliflozin treatment led to increases in cyst volume and kidney weight, suggesting that SGLT2 inhibition may have complex effects in ADPKD [121]. The EMPA-PKD trial is a randomized, double-blind, placebo-controlled clinical study designed to evaluate the safety and potential efficacy of empagliflozin, an SGLT2 inhibitor, in patients with rapidly progressive ADPKD [122].

#### 4.2.3. Targeting Glutamine and Amino Acid Metabolism

Glutaminase (GLS) inhibitors, such as telaglenastat (CB-839), can force cyst cells into glutamine starvation. Podrini et al. [32] revealed that *PKD1*-mutant cells preferentially utilize glutamine to fuel the TCA cycle and sustain fatty acid synthesis; interfering with glutamine uptake or conversion significantly retarded cell growth and survival. Moreover, the silencing of ASNS was lethal in *PKD1*-mutant cells when combined with glucose deprivation.

Similarly, cystic epithelial cells exhibit metabolic reprogramming that includes a dependency on exogenous arginine due to diminished expression of argininosuccinate synthetase 1 (ASS1), the enzyme responsible for endogenous arginine synthesis; this arginine auxotrophy renders these cells susceptible to arginine deprivation strategies. In oncology, arginine deprivation therapy has been investigated in tumors deficient in ASS1 expression [123], a therapeutic approach that exploits the metabolic weakness of ASS1-deficient cells, which are unable to synthesize arginine and, thus, depend on extracellular sources. Given the observed downregulation of ASS1 in ADPKD cyst-lining cells, similar to clear-cell renal cell carcinoma, arginine deprivation could potentially impair cyst growth and progression. The researchers demonstrated that arginine deprivation, or inhibition of Arg1, significantly attenuated cystogenesis both in vitro and in murine models [124]. The rationale is that lowering arginine availability impairs polyamine synthesis and mTOR signaling in cyst-lining cells.

Asparagine metabolism has garnered attention due to its role in cellular proliferation and survival. Emerging evidence showed elevated plasma asparagine levels in pediatric and young adult ADPKD patients compared with healthy controls, indicating a potential alteration in asparagine metabolism associated with disease progression [64]. Additionally, research by Steidl et al. demonstrated that ASNS localizes to the base of primary cilia and mediates glutamine-dependent mitochondrial anaplerosis during nutrient stress, suggesting a link between asparagine metabolism and ciliary function in cystic epithelial cells [125]. In oncology, L-asparaginase has been effectively utilized to deplete extracellular asparagine; this deficiency renders them dependent on extracellular asparagine, and depletion of this amino acid by L-asparaginase leads to selective apoptosis of leukemic blasts [126]. While the application of asparaginase in ADPKD remains speculative and untested, the metabolic parallels between cystic epithelial cells and certain cancer cells provide a rationale for exploring asparagine depletion strategies. Such interventions could potentially disrupt the metabolic dependencies of cystic cells, offering a novel angle distinct from traditional signaling inhibitors; however, further research is necessary to elucidate the role of asparagine metabolism in ADPKD, and to assess the feasibility and safety of asparaginase-based therapies in this context.

#### 4.2.4. Inhibitors of Signaling with Metabolic Impact

##### mTOR Inhibitors

Drugs including rapamycin (sirolimus) and everolimus directly inhibit mTORC1. While they showed efficacy in animal models, reducing cyst growth by curtailing cell proliferation and anabolic metabolism, human trials in ADPKD were disappointing in terms of slowing kidney growth, likely due to insufficient drug delivery to cysts, compensatory pathway activation, and off-target effects.

Shillingford et al. demonstrated that mTOR inhibition with rapamycin effectively reduced cyst growth and preserved renal function in *PKD1*-deficient mouse models, indicating preclinical efficacy [127]. A randomized controlled trial was conducted to assess sirolimus in ADPKD patients, and no significant benefit in slowing kidney growth or preserving renal function was found compared with placebo [128]. Walz et al. reported that, in a double-blind trial, everolimus modestly reduced kidney volume increase, but did not significantly impact renal function decline, highlighting limited clinical efficacy [129]. Liu et al. reported that mTOR inhibition alone may activate compensatory pathways. Dual mTOR/PI3K inhibition with NVP-BEZ235 abrogated pro-proliferative signals and normalized kidney morphology and function by blocking proliferation and fibrosis. Therefore, multi-target PI3K/mTOR inhibition could be more effective in treating ADPKD [130]. Freedman et al. introduced AV457, a new rapamycin analog, which demonstrated comparable efficacy to everolimus, with improved safety in human kidney organoids, suggesting a potential for better clinical outcomes [131].

##### Somatostatin Analogues

The therapeutic application of somatostatin analogues, such as octreotide and lanreotide, in ADPKD has been investigated due to their capacity to inhibit adenylate cyclase via somatostatin receptors, thereby reducing cAMP levels; this reduction is associated with decreased cystogenesis and modulation of metabolic enzyme expression. The outcomes of clinical trials have demonstrated that these agents can modestly slow cyst growth, particularly in hepatic manifestations of the disease.

Van Keimpema et al. reported that lanreotide treatment over 24 weeks resulted in a significant reduction in liver volume in patients with polycystic liver disease, including those with ADPKD, compared with placebo [132]. While lanreotide slowed kidney growth, it did not significantly affect the rate of decline in eGFR [133].

In 2019, Perico et al. reported a randomized, double-blind, placebo-controlled, multicenter trial; in this phase III trial, octreotide-LAR was evaluated in ADPKD patients with chronic kidney disease stages 3b to 4. Over three years, octreotide-LAR significantly slowed kidney volume growth and delayed the progression to end-stage renal disease, particularly in patients with stage 4 CKD [134].

The outcomes of these studies collectively support the viewpoint that somatostatin analogues, by inhibiting cAMP production, can modestly slow cyst growth in ADPKD, particularly affecting liver cysts; however, their impact on kidney function decline appears limited, indicating the need for further research to optimize therapeutic strategies.

##### HIF-1α Inhibitors

The hypothesis that inhibiting hypoxia-inducible factor 1-alpha (HIF-1α) could normalize the metabolic profile of cystic epithelial cells in ADPKD is supported by the results of several preclinical studies, which elucidate the role of HIF-1α in promoting glycolysis and cyst expansion, suggesting that targeting this pathway may offer therapeutic benefits. Kraus et al. demonstrated that HIF-1α expression is upregulated in the cystic kidneys of *PKD1*-deficient mice. Genetic deletion of Hif-1α significantly attenuated cyst growth, indicating its role in disease progression [68]. The authors of another study found that HIF-1α activation leads to increased chloride secretion in cystic epithelial cells, contributing to cyst enlargement [71]. Courtney et al. reported that PT2385, a HIF-2α antagonist, has a favorable safety profile and is active in patients with previously heavily pretreated clear-cell renal cell carcinoma (ccRCC), validating direct HIF-2α antagonism for the treatment of patients with ccRCC [135]. While PT2385 targets HIF-2α and is studied in cancer contexts, its development underscores the therapeutic potential of HIF pathway inhibition, which may be extrapolated to HIF-1α in ADPKD. The outcomes of the aforementioned studies suggest that HIF-1α plays a significant role in the metabolic alterations and cyst growth observed in ADPKD. Although direct clinical trials targeting HIF-1α in ADPKD are lacking, the preclinical evidence provides a compelling rationale for exploring HIF-1α inhibitors as potential therapeutic agents in this disease context.

Recent efforts have been focused on the therapeutic modulation of metabolic pathways implicated in cyst growth and expansion in ADPKD, including caloric restriction, AMPK activation, glycolysis inhibition, and glutamine metabolism targeting, among others. Table 1 provides a comprehensive summary of the key therapeutic strategies targeting metabolic reprogramming in ADPKD.

## 5. Discussion and Future Directions

In recent studies, researchers have determined that metabolic reprogramming is not just an epiphenomenon but a key driver of the cystogenesis of ADPKD. Loss-of-function *PKD1* or *PKD2* mutations cause the dysregulation of polycystin-1/2 function, leading to a cascade of metabolic changes. Metabolic reprogramming in ADPKD encompasses several hallmark alterations, the characteristics of which include enhanced aerobic glycolysis, increased glutamine utilization, and decreased oxidative phosphorylation; these metabolic changes are not only the result of cyst formation but also actively promote the occurrence of cysts [32]. Multiple signaling pathways are involved in the formation of a metabolic environment conducive to uncontrolled cell proliferation and cyst expansion, such as cAMP, PKA, mTORC1, and c-Myc. The associated metabolic changes not only facilitate the formation and growth of cysts but also have the potential to impact the overall metabolic state and comorbid conditions in patients with ADPKD.

ADPKD is a hereditary disorder characterized not only by renal cyst development but also by a spectrum of systemic comorbidities that significantly impact patient morbidity and mortality. Hypertension is a prevalent early manifestation, often preceding detectable declines in renal function; this early-onset hypertension is attributed to both renal and vascular abnormalities, including impaired endothelial function due to polycystin deficiency. Recent evidence has demonstrated that *PKD2* is expressed in arterial endothelial cells, where it acts as a calcium-permeable channel that opens in response to laminar shear stress, allowing Ca^2+^ influx into endothelial cells [26]. *PKD2* activation by flow leads to nitric oxide (NO)-mediated vasodilation. Mice with endothelial-specific deletion of *PKD2 (PKD2*^KO)^ showed impaired flow-mediated dilation, reduced NO production, and significantly elevated systemic blood pressure, without altered cardiac function or kidney anatomy. Building upon these findings, MacKay et al. conducted a study to further describe the functional architecture of the PC1 and PC2 complexes in vascular endothelial cells. The authors demonstrated that PC1 and PC2 form a heteromeric ion channel complex at the plasma membrane, functioning as a shear stress-responsive mechanosensor that promotes calcium influx and vasodilatory signaling. Importantly, the endothelial-specific deletion of *PKD1* or *PKD2* resulted in impaired acetylcholine-induced vasodilation, elevated systemic blood pressure, and reduced expression of flow-responsive genes such as *KLF2*, *NOS3*, and *COX2* [27].

Polycystins occur in smooth muscle, vascular endothelium, cardiomyocytes, and fibroblasts, and they are associated with vascular abnormalities. Endothelial dysfunction is an early feature of vascular damage in ADPKD patients, followed by a stiffening of the large elastic arteries and increased pulse wave velocity.

Furthermore, obesity is known to be independently associated with the rate of ADPKD progression [136]. ADPKD patients exhibit a higher incidence of insulin resistance and type 2 diabetes mellitus, which are linked to the defective glucose metabolism and metabolic reprogramming observed in ADPKD models [137]. The coexistence of diabetes mellitus in ADPKD patients is associated with increased total kidney volume and earlier onset of hypertension, suggesting a synergistic effect on disease progression [137]. The results of the aforementioned studies indicate that the metabolic reprogramming of ADPKD, characterized by increased glycolysis and impaired fatty acid oxidation, may exacerbate these comorbidities.

Therefore, cardiovascular and metabolic comorbidities, including hypertension, left ventricular hypertrophy, valvular complications, type 2 diabetes mellitus, and dyslipidemia, are more prevalent among patients with autosomal dominant polycystic kidney disease (ADPKD) [138,139]. Moreover, they are significantly associated with a substantial increase in the risk of all-cause mortality [140].

Therapeutic strategies targeting metabolic pathways have demonstrated potential efficacy in ADPKD; however, their implications for comorbidities must be meticulously assessed.

Emerging metabolic interventions, including ketogenic diets and pharmacologic agents, such as metformin, aim to target the altered energy metabolism characteristic of cystic epithelial cells. In preclinical studies, therapeutic approaches targeting abnormal metabolic reprogramming have shown potential results. In early-phase clinical trials, it has been shown that pharmacological agents, such as metformin, can slow disease progression in ADPKD patients. In a preclinical research study, authors assessed the combined effects of tolvaptan and salsalate in a *PKD1* mouse model [116], and the results showed that the combined treatment has a synergistic effect in reducing renal injury, cell proliferation, inflammation, and fibrosis, in addition to improving mitochondrial health and antioxidant responses. The results of the aforementioned studies suggest that the concurrent use of vasopressin V2 receptor antagonists, such as tolvaptan, alongside metabolic modulators, offers the potential to simultaneously suppress aberrant cAMP signaling and correct underlying metabolic dysfunction. Furthermore, early dietary interventions also hold promise; current research suggests that altering the body’s energy metabolism through calorie restriction and a ketogenic diet may delay the occurrence or progression of cyst formation.

While these strategies hold promise for slowing cyst growth and preserving renal function, their systemic effects necessitate careful evaluation, including on vascular tone, endothelial metabolism, and redox balance. For instance, caloric restriction or ketogenic diets may confer cardiovascular benefits through enhanced endothelial mitochondrial function and reduced oxidative stress, yet prolonged ketosis may exacerbate dyslipidemia or increase vascular stiffness in predisposed individuals [141]. Similarly, pharmacological agents, such as metformin, may improve both metabolic and vascular homeostasis via AMPK activation, but their effects on endothelial polycystin signaling remain to be elucidated [142]. Overall, these findings highlight the dual potential of metabolic therapy in ADPKD, not only in limiting cystogenesis but also in modifying comorbidities, such as hypertension, endothelial dysfunction, and metabolic syndrome.

Although metabolic transformation therapy is attractive, some problems increase the difficulty of its translation into clinical settings. Patient adherence to restrictive diets or long-term metabolic therapies poses a significant barrier. Moreover, these interventions have wide-ranging impacts and require a precise medical approach to explain the interplay between renal and extrarenal disease mechanisms. Concerns regarding the chronic safety profiles of metabolic inhibitors and the variability in individual patient responses underscore the need for rigorous, controlled clinical trials; therefore, future research should not only monitor renal prognosis, but also cardiovascular and metabolic changes, in order to ensure a comprehensive assessment of the benefit–risk profile of ADPKD patients; only through such studies can these emerging interventions be validated and integrated into standard-of-care regimens for ADPKD.

Furthermore, how metabolic alterations in cystic epithelial cells influence and are influenced by other renal cell types, including fibroblasts and immune cells, remains to be elucidated. Investigating immune–metabolic crosstalk, such as the role of arginase-positive macrophages (M2-like macrophages) in promoting cyst growth, may reveal novel therapeutic targets.

Targeting cellular metabolism represents a paradigm shift in the management of ADPKD, aligning nephrology with concepts from oncology and metabolic disease. While current therapies primarily focus on supportive measures and vasopressin antagonism, emerging metabolic interventions offer promising avenues for disease modification; the consistency of metabolic alterations across various models and the initial positive signals from clinical studies are encouraging. With continued research and clinical validation, future therapies may involve a multifaceted approach. Current research suggests that combining metabolic intervention with existing treatment methods and lifestyle changes may alter the therapeutic prospects of ADPKD.

## Figures and Tables

**Table 1:** Therapeutic strategies targeting metabolic reprogramming in ADPKD.

Therapeutic Strategy	Metabolic Target	Drug/Intervention	Reference
Caloric Restriction and Fasting	Induce ketosis, inhibit mTOR, activate AMPK	Fasting, TRF, CR	[91,92,93,94,95,96,97,98]
Ketogenic Diet and Ketone Supplementation	Sustain ketosis, inhibit glycolysis, activate AMPK	Ketogenic diet, β-hydroxybutyrate (BHB), citrate supplementation	[99,100,101,102]
Protein or Amino Acid Restriction	Reduce amino acid availability (glutamine, arginine)	Low-protein diet, BPTES (glutaminase inhibitor)	[40,103,104,105]
Hydration and Caffeine Avoidance	Reduce vasopressin and cAMP levels	Increased water intake, caffeine avoidance	[106,107,108,109]
AMPK Activators (Metformin, Salsalate)	Activate AMPK, inhibit mTOR, improve mitochondrial function	Metformin, salsalate	[111,112,113,114,115,116]
Glycolysis Inhibition (2DG, SGLT Inhibitors)	Inhibit glycolysis, reduce ATP supply to cyst cells	2DG, Phlorizin, dapagliflozin, empagliflozin	[117,118,119,120,121,122]
Arginine Deprivation	Reduce polyamine synthesis, inhibit mTOR signaling	Arginase inhibitors	[124]
Asparagine Targeting	Disrupt glutamine-derived asparagine metabolism	L-asparaginase (theoretical)	[64,125,126]
mTOR Inhibitors (Rapamycin, Everolimus)	Inhibit mTORC1 signaling, suppress cell growth	Rapamycin, sirolimus, everolimus, NVP-BEZ235, AV457	[127,128,129,130,131]
Somatostatin Analogues (Octreotide, Lanreotide)	Suppress cAMP production	Octreotide-LAR, lanreotide	[132,133,134]
HIF-1α Inhibitors	Suppress glycolysis and cystic proliferation	HIF-1α genetic deletion, HIF inhibitors (PT2385 for HIF-2α)	[68,71,135]

## Data Availability

Data are available upon request.
